# Serology supportive of recent coxsackievirus B infection is correlated with multisystem inflammatory syndrome in children (MIS-C)

**DOI:** 10.1128/spectrum.01741-24

**Published:** 2025-02-05

**Authors:** Ramesh Kordi, Arthur J. Chang, Mark D. Hicar

**Affiliations:** 1 Department of Pediatrics, Division of Infectious Diseases, State University of New York at Buffalo, Buffalo, New York, USA; 2 Division of Pediatric Infectious Diseases, University of Nebraska Medical Center, Omaha, Nebraska, USA; Brown University, Providence, Rhode Island, USA

**Keywords:** coxsackievirus A, coxsackievirus B, complement fixation test, enterovirus, immunofluorescence assay, sero-epidemiology, multisystem inflammatory syndrome in children, MIS-C

## Abstract

Rarely, severe acute respiratory syndrome coronavirus 2 (SARS-CoV-2) will lead to myocarditis associated with multisystem inflammatory syndrome in children (MIS-C). It remains unclear why MIS-C only targets specific children. To explore an association between coxsackievirus infections with MIS-C, we investigated the sero-epidemiology of CV in admitted pediatric patients in relation to the pandemic. This retrospective case–control study was performed by chart review of children (age ≤21 years) admitted to a tertiary care hospital with CV serological testing from January 2017 to August 2023. Clinical, laboratory, and imaging findings were used to classify patients as MIS-C and CV-unlikely or CV-possible for non-MIS-C patients. Out of 182 admissions (179 patients, median age, 6), CVB complement fixation (CF) assay on serotypes B1–B6 and CVA immunofluorescence assay IgG on serotypes A7, A9, A16, and A24 were positive in at least one serotype in 59.2% and 80.7% of cases, respectively. We observed a significant drop in CVB CF seropositivity during the peak of social distancing in 2020. The likelihood of elevated CVB CF titers was significantly higher in MIS-C than the CV-unlikely group (OR: 1.92, 95% CI: 1.02–3.63, *P*: 0.04) and showed a trend toward higher values in African Americans than Whites (OR: 1.57, 95% CI: 0.98–2.50, *P*: 0.057). The frequency of MIS-C was considerably higher in African Americans than Whites (18.1% versus 9%, *P*: 0.1). A higher likelihood of elevated CVB CF titers in patients with MIS-C compared with those unlikely to have acute CV infection along with a relatively higher frequency of MIS-C in African Americans warrants further investigation into the role of CVB infection in MIS-C development.

**IMPORTANCE:**

The emergence of multisystem inflammatory syndrome in children (MIS-C) during the SARS-CoV-2 pandemic raised major concerns in providers caring for children. This condition presents a hyper-inflammation state that can lead to severe complications, including myocarditis and cardiogenic shock. The pathogenesis of MIS-C has not been fully understood. Understanding the pathogenesis of this condition is not only important for developing effective treatments but also for applying preventive strategies. A two-hit hypothesis leading to MIS-C has been proposed. Coxsackievirus infections are prevalent during childhood and can also cause myocarditis, and coxsackievirus B specifically has been shown to cause persistent RNA presence in host cells, leading to continued inflammation. Herein, we show that elevated coxsackievirus B titers are associated with MIS-C cases, implying a role of successive infections with these viruses contributing to such a hyperinflammatory state. This study supports the need for larger investigations into this association.

## INTRODUCTION

Multisystem inflammatory syndrome in children (MIS-C) characterized by a hyper-inflammation state generally occurs 2–6 weeks after a SARS-CoV-2 infection in children (1–3). Multiple organ systems are affected during MIS-C, including the most striking finding of cardiac dysfunction in approximately 40% of patients (4, 5). In fact, the condition was first recognized as a collection of children with myocarditis and having a Kawasaki-like illness (6–8). The precise pathogenesis of MIS-C remains unclear; however, the role of an earlier infection as a secondary priming agent has been suggested (9, 10). Studies investigating the involvement of other pathogens, such as cytomegalovirus and Epstein–Barr virus have so far been inconclusive (11, 12). Given the similarities between manifestations of MIS-C and coxsackievirus (CV) infections, including fever, skin rash, mucositis, and conjunctivitis, many patients with a final diagnosis of MIS-C had CV testing during their admission with a number of these returning positive results.

The single-stranded RNA genus *Enterovirus* includes enteroviruses, CVs, rhinoviruses, polioviruses, and echoviruses. Among 10 true enteroviruses (EV A–J), EV species of A–D are associated with human infections and include more than 100 EV serotypes, including CVA, CVB, and numbered enteroviruses, like EV-D68 and EV-A71 (13, 14). These viruses cause a spectrum of clinical manifestations ranging from mild presentations, such as fever, skin rash, oral mucositis (i.e., hand, foot, and mouth disease), upper respiratory manifestations, and gastroenteritis, to severe even life-threatening conditions, particularly in infants, young children, and immunocompromised individuals, including myocarditis (15, 16), pneumonitis (17), flaccid myelitis (18, 19), and encephalitis (20, 21). Severe outbreaks of CVB-associated myocarditis (22) and meningoencephalitis (23, 24) have been documented. CVB-induced myocarditis may progress to dilated cardiomyopathy (25). Epidemiological, clinical, and experimental studies show a strong connection between past CVB infections and certain autoimmune diseases, such as type I diabetes (26, 27). The presence of persistent CVB RNA remnants causing a persistent immune response in tissues and the development of autoantibodies has been proposed as a potential cause of some CVB-induced chronic diseases (28, 29). Thus, preceding CVB infections are a legitimate area of investigation in MIS-C pathogenesis. Additionally, during clinical care, we observed that several children with MIS-C had positive CV titers. Our current study formally explored the clinical correlations in children tested for CVB and CVA serology and compared the results in three clinical categories: MIS-C and the non-MIS-C groups of CV-unlikely and CV-possible.

## MATERIALS AND METHODS

We performed a retrospective case–control study approved by the University at Buffalo IRB (# STUDY00007590). The inclusion criteria included patients admitted to Oshei Children’s Hospital ≤21 years of age who had serological tests ordered for CVA (charge activity codes of 10004309 to 10004314) and/or CVB (charge activity codes of 10004315 to 10004320) from January 2017 to August 2023. There were 187 admissions from 184 patients with CV serological tests. We excluded two cases from patients who were older than 21 years old (32 and 34 years) and three cases in whom CV serological tests were ordered, but results were not available for undisclosed reasons.

### Data collection and categorization

For patients who were re-admitted during the study period, the results of each admission were recorded and evaluated. Therefore, we referred to each admission as a “case.” We collected data regarding patients’ demographics, vital signs, symptoms and physical examination upon admission. Available imaging results, echocardiography, and specific laboratory markers were collected, including complete blood count, erythrocyte sedimentation rate (ESR), c-reactive protein (CRP), ferritin, procalcitonin, cardiac markers [Troponin I, B-type natriuretic peptide (BNP)], complete metabolic panel, cultures, and bacterial/viral testing (serology and PCR). Per the manufacturer’s testing guideline (LabCorp, Burlington, NC, USA), positive result cutoffs for CV serology were as follows: CVA IgG immunofluorescence assay (IFA) 1:100, CVA complement fixation (CF) 1:8, and CVB CF 1:8. In patients who were re-admitted, we considered the CV serological results in both admissions for the analysis.

Respiratory viral panel PCR was performed using the Panther Fusion System platform (by Hologic). Specific tests included those for influenza viruses and respiratory syncytial virus (Flu A/B/RSV); adenovirus, human metapneumovirus, and rhinoviruses (AdV/hMPV/RV); and parainfluenza 1–4 (Paraflu). Through its multiplex real-time PCR (RT-PCR) assays using specific primers and probes that target unique genetic sequence of each virus, this system can accurately identify and differentiate each virus. Notably, the rhinovirus test has no cross-reaction to tested enteroviruses, which can differentiate it from enteroviruses. From 2021, for some patients, based on their clinical symptoms, a multiplex real-time test (by Cepheid) was performed to detect SARS-CoV-2, influenza A/B, and RSV. All serological and molecular tests were performed before intravenous immunoglobulin (IVIG) administration in patients who required it.

We also reviewed the notes from the primary team and consultation notes from various disciplines, including infectious diseases, rheumatology, cardiology, nephrology, gastroenterology, immunology, and endocrinology. Patients with MIS-C diagnosis were re-confirmed based on updated criteria developed by The Council of State and Territorial Epidemiologists (CSTE) and CDC (30). We classified patients as “CV-unlikely” if they met any of these criteria: (i) a documented bacterial infection (without any concomitant viral disease) based on cultures and/or clinical findings, (ii) a documented acute viral infection other than CVs based on serological and or polymerase chain reaction (PCR) findings, (iii) a non-infectious condition based on clinical, laboratory, and imaging findings. Patients with clinical and/or laboratory results consistent with a viral syndrome, but without a documented viral pathogen (unspecified viral infection), or with a positive enterovirus RT-PCR were classified as CV-possible. CV serological test results were intentionally not used in the classification of cases into these groups. Thus, we had three groups of cases: (i) MIS-C diagnosis and the two non-MIS-C groups, (ii) CV-unlikely, and (iii) CV-possible.

### Statistical methods

The data were analyzed using SPSS 29 software (SPSS Inc., Chicago, IL, USA). For comparing CV seropositivity between groups, we used the proportional odds model (also known as ordinal logistic regression model). Through this model, the serological test results in groups are left as original form as ordinal levels of antibody titers without defining a cut-off (31). The χ test was also utilized to compare seropositivity rates between groups by defining a cut-off level. We considered *P*-values less than 0.05 as statistically significant.

## RESULTS

We analyzed the medical records from 182 eligible admissions in 179 patients with a median age of 6 years (3 weeks to 20 years) and a male-to-female ratio of 96:83. Only three patients had repeat testing from hospitalizations during this period. The race of patients was as follows: White, 111 (62%); African American, 44 (24.6%); Asian, 7 (3.9%); Asian Indian, 5 (2.8%); unspecified, 12 (6.7%).

There were 167 cases with CVB serological tests for serotypes B1–B6 through the complement fixation (CF) method. Eighteen cases underwent CVA serological tests via CF method from January 2017 to August 2018 for serotypes A2, A7, A9, A10, and A16. Following August 2018, CVA serological tests were performed through immunofluorescence assay (IFA) IgG on serotypes A7, A9, A16, and A24 in 150 cases. Both CVB CF and CVA IFA IgG serological tests were performed in 138 (75.8%) cases. Fever was the most common presenting symptom (73.7%), followed by skin rash (37.9%), gastrointestinal (34%), respiratory (31%), neurological symptoms (20%), oral mucositis (14.5%), and conjunctivitis (11.1%).

Out of 98 cases (53.8%) who were clinically diagnosed with a viral syndrome, eight had superimposed bacterial infections. Only 37 (59.6%) of these 98 cases had a viral pathogen identified by serology and/or PCR (Table S1). The characteristics of cases with bacterial infections (29 cases, including eight with concomitant viral infections) and non-infectious conditions (44 cases) are illustrated in Tables S2 and S3, respectively. Overall, 42 cases had enterovirus (EV) RT-PCR test in their medical records, including 22 nasopharyngeal EV RT-PCR only, 20 EV CSF RT-PCR only, and three had the test on both specimens. Among all these patients, only one was positive in the nasopharyngeal specimen. The details of 42 patients with enterovirus molecular testing are revealed in Table S4.

### Coxsackievirus B testing results

**Fig 1:**
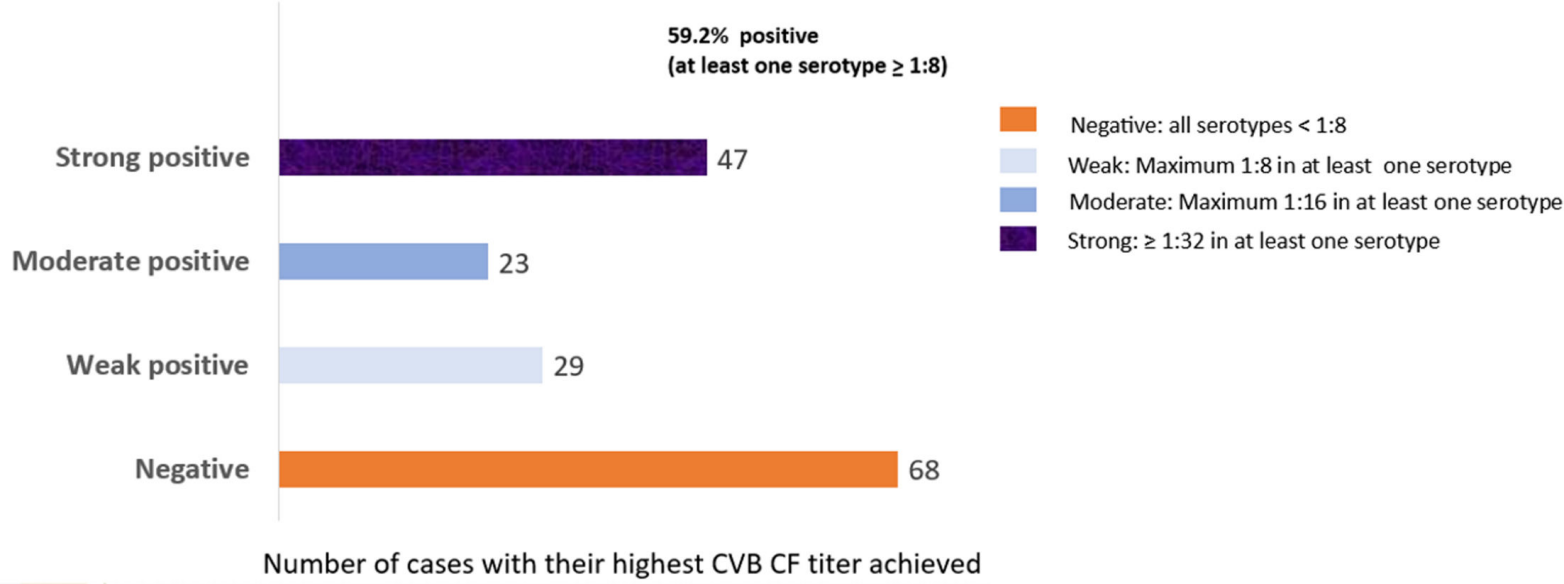
The number of cases with their maximum CVB CF titer in at least one serotype among 167 admissions in Oishei Children’s Hospital.



Among the 167 cases with CVB serological test through the complement fixation method, a positive result (≥1:8) was detected for at least one serotype in 99 cases (59.2%) (Fig. 1), with a median number of positive serotypes of 4 (range, 1–6). The highest positivity rate was shown for B5 serotype (79/167, 47.3%). The positivity rate for other serotypes ranged from 26.3% for B3 to 35.9% for B1. The B5 serotype had also the highest rate of strong positivity (titer ≥1:32) among all serotypes (37/167, 22.1%).

 The overall CVB CF positivity rate was the lowest in ages younger than 1 year old (5/16, 31.3%), with similar elevations in groups older than 1 year of age: seropositivity ages 1–4 years (29/47, 61.7%), 5–12 years (35/56, 64.2%), and ≥13 years (29/48, 60.4%).

**Fig 2:**
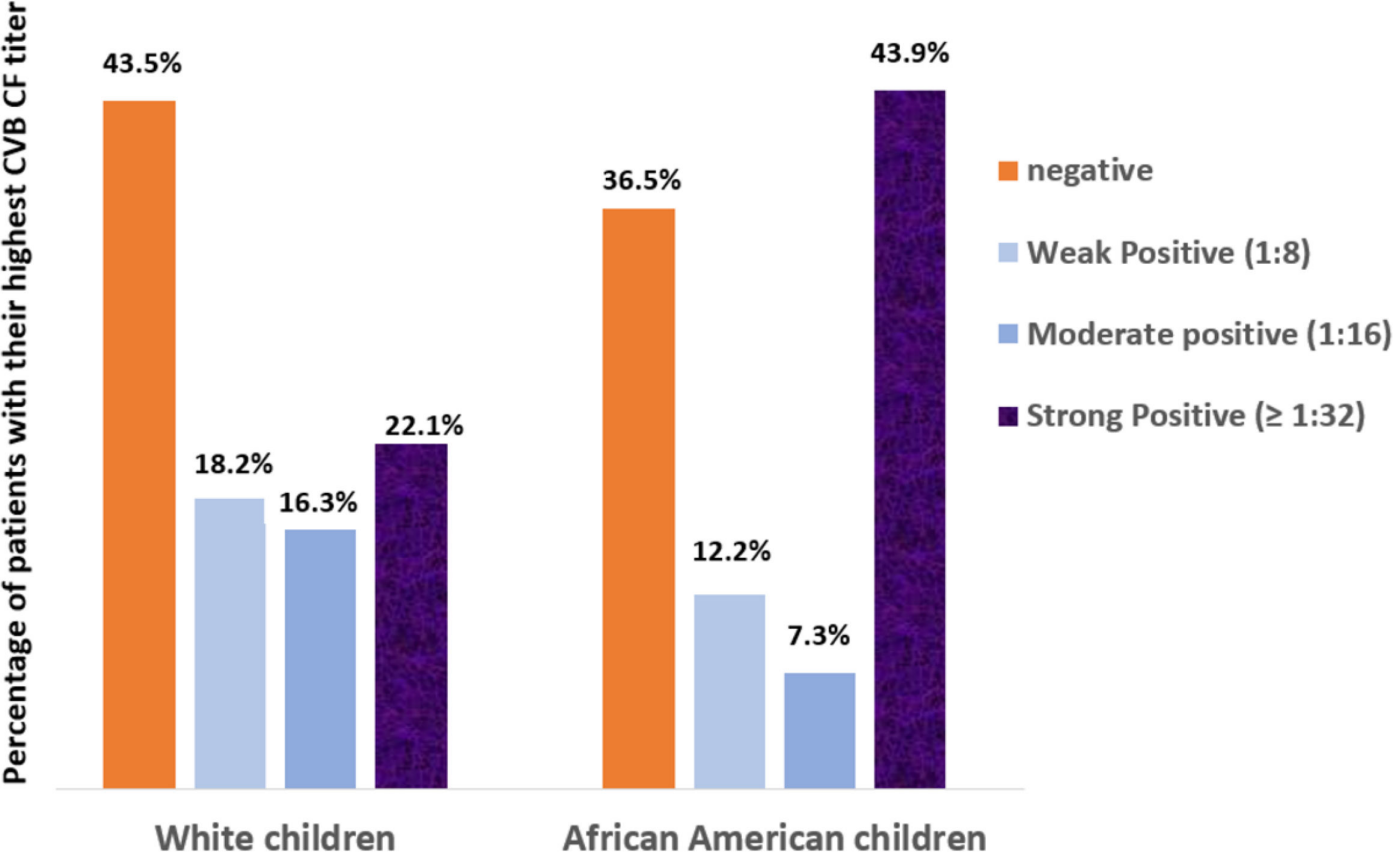
CVB CF positivity rate of highest titer achieved for serotypes B1–B6 among White children (total 104 patients) versus African American children (total, 41 patients).

There were 104 White and 41 African American cases who had CVB CF testing in their charts. The likelihood of higher CVB CF titers was notably higher in African American than White children but did not reach statistical significance (odds ratio: 1.57, 95% CI: 0.98–2.50, *P*: 0.057). However, the frequency of strong (titer ≥1:32) CVB CF positivity rate was significantly higher in African Americans than Whites (18/41, 43.9%, vs 23/104, 22.1%, *P* = 0.01) (Fig. 2).

**Fig 3:**
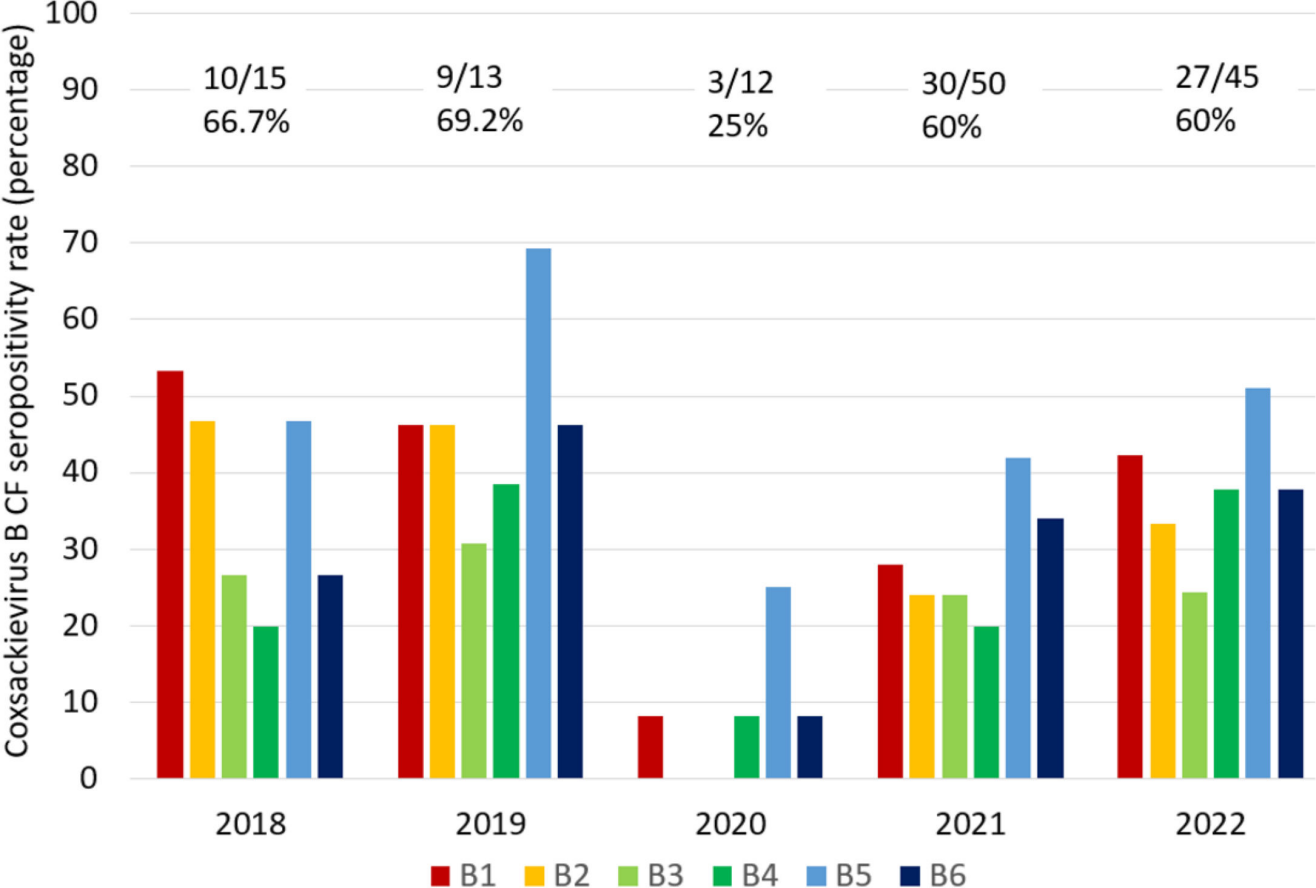
The CVB CF seropositivity rate(titers ≥ 1:8) for various serotypes during the years 2018–2022. The floating numbers show the seropositivity rate (the number of patients with a positive test in at least one serotype to the total number of tested individuals) according to the year.

We found a significant decline in CVB CF seropositivity in 2020 during the SARS-CoV-2 pandemic. The CVB CF seropositivity rate (titer ≥1:8) was significantly lower in 2020 as compared with 2019 (3/12, 25% vs 9/13, 69/2%; *P* = 0.047) (Fig. 3).

### Coxsackievirus A testing results

There were 18 CVA serological tests through the complement fixation (CF) method from January 2017 to August 2018, of which two (11%) had positive results. One case was an 18-year-old male with positive A4 serotype at 1:64, and another case was a 10-year old male who was positive for CVA serotypes A4: 1:64, A9; 1:32, A7, A10, and A16; 1:16. Both patients were diagnosed with unspecified viral infections.

Since September 2018, all CVA serological tests have been performed via indirect immunofluorescence assay (IFA) IgG on A7, A9, A16, and A24. There were 150 cases with CVA IFA IgG serological test, of which 121 (80.7%) were positive for at least one serotype (titer ≥1:100). Among positive cases, 116 (95.8%) were positive for all serotypes. The positivity rate for each serotype ranged from 77.2% for A7 to 80.5% for A24. The seropositivity rate for at least one serotype increased from 61.5% in ages less than 1 year old to the highest level at 90.2% in the age group of 1–4 years old. We did not observe a significant difference in CVA IFA IgG seropositivity rate from 2018 to 2022. The CVA seropositivity rate for at least one serotype through the years was as follows: 2018, 5/6 (83.3%); 2019, 11/14 (78.6%); 2020, 11/13 (84.6%); 2021, 41/48 (85.4%); 2022, 16/21 (76.2%).

The likelihood of higher CVA IFA titers was relatively higher in African Americans than in White children (odds ratio: 1.36: 95% CI: 0.67–2.76, *P*: 0.39). The strong positivity rate (titer, 1:1,600) was almost equal between the two groups (10/33, 30.3%, vs 27/94, 28.7%, *P*: 0.86).

### Repeat admissions

Three patients had repeat testing in intervals of 3, 34, and 35 months (Table S5). The first case was a 5-year-old female initially admitted with fever, cough, and widespread maculopapular skin rash who was positive for CVB CF B5 at 1:8 with no other detected viral etiology. She was re-admitted 3 months later with pancreatitis due to hypertriglyceridemia. At that time, repeat testing showed a fourfold rise in CVB CF B5 titer to 1:32, supportive of the initial illness being CVB B5. The second child was a 9-year-old male who was initially diagnosed with Stevens–Johnson Syndrome and was CVB CF positive for all serotypes, with B5 having the highest level at 1:32. He was also positive for all CVA IFA serotypes with the highest level for A16 at 1:200. He was re-admitted 34 months later with re-appearance of oral and genital lesions and sore throat. The investigation on a viral agent including HSV1/2 PCR from oral lesions was negative. The final diagnosis was recurrent Stevens–Johnson Syndrome. CVA IFA titers remained unchanged, and CVB CF titers were completely negative for all serotypes. The third case was a 15-year-old female with scleroderma who had a diagnosis of acute viral myocarditis with ejection fraction of 40% on the first admission. A cardiac biopsy was not performed, and there were no other viral studies supportive of a specific diagnosis. CVB CF and CVA IFA IgG testing was negative at this time. In our medical record, we did not have specific repeat convalescent testing results obtained from the months that followed the initial admission, which could have assisted with the diagnosis of the myocarditis. After 37 months, she was admitted for SARS-CoV-2 pneumonia with fever, chest pain, shortness of breath, respiratory failure, and cardiogenic shock. At that time, CVB CF was positive for B1 and B3–B6, with the highest level for B5 at 1:16. CVA IFA IgG had also turned positive for all serotypes with A24 at the highest level of 1:400.

### Admissions with the final diagnosis of MIS-C

**Fig 4:**
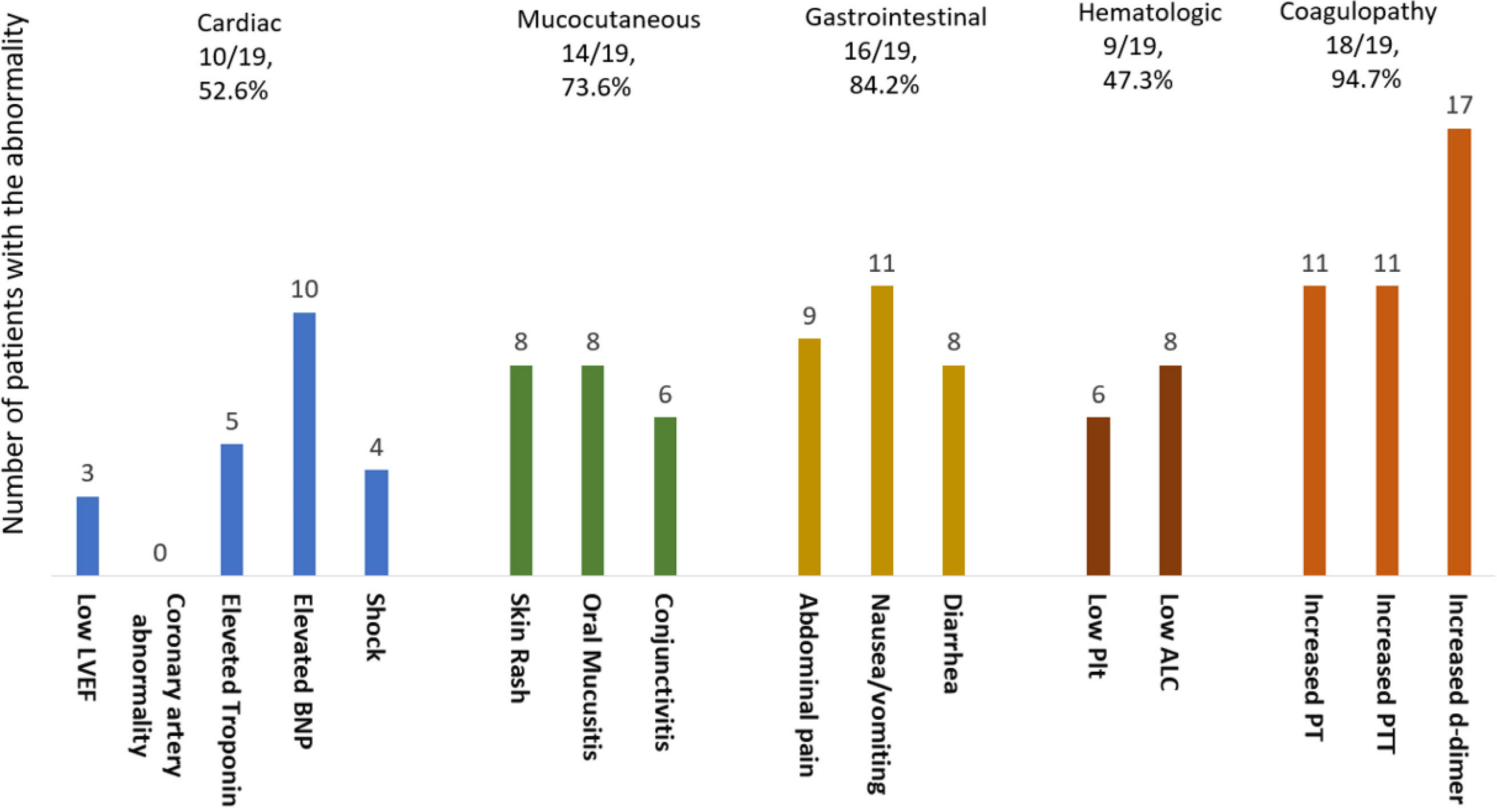
The frequency of laboratory abnormality, clinical symptoms, and organ involvement in 19 patients with MIS-C diagnosis. The floating numbers represent the rate of laboratory/clinical manifestations. Low left ventricular ejection fraction (LVEF): <55%, elevated troponin: >0.04, elevated B-type natriuretic peptide (BNP: >100 pg/mL), low platelet (Plt): <150,000/μL, low absolute lymphocyte count (ALC): <1,000/μL, increased prothrombin time (PT): >13.5 s, increased partial thromboplastin time (PTT): >35 s.

There were 19 cases with the final diagnosis of MIS-C, with a median age of 6 years (9 months to 17 years) and a male-to-female ratio of 13:6 (2.16). There were 10 Whites and eight African Americans, and in one case, the race was not specified. The frequency of MIS-C among African Americans was notably higher than Whites but not statistically significant (8/44, 18.1%, versus 10/111, 9%, *P*: 0.1). Our MIS-C cases who had serological coxsackievirus tests were admitted from January 2021 to May 2021 (nine patients) and from November 2021 to March 2022 (10 cases). The clinical, laboratory characteristics, and coxsackievirus serological test results of patients with MIS-C are illustrated in Fig. 4; Tables S6 to S8.

### MIS-C, CV-unlikely, and CV-possible groups and CV serological results

Among 182 admissions, we had 19 MIS-C cases (10.4%), 101 CV-unlikely (55.4%), and 62 CV-possible cases (34%) including one patient with positive enterovirus RT-PCR from the nasopharyngeal specimen. Moreover, 18 out of 19 patients with MIS-C had CVB CF testing in their file. The median age of CV-unlikely and CV-possible groups was 6 years (range: 1 month to 20 years) and 6.5 years (range: 1 month to 19 years), respectively. Among 167 cases with CVB CF test in their charts, the MIS-C, CV-unlikely, and CV-possible groups had 18, 91, and 58 cases respectively.

**Fig 5:**
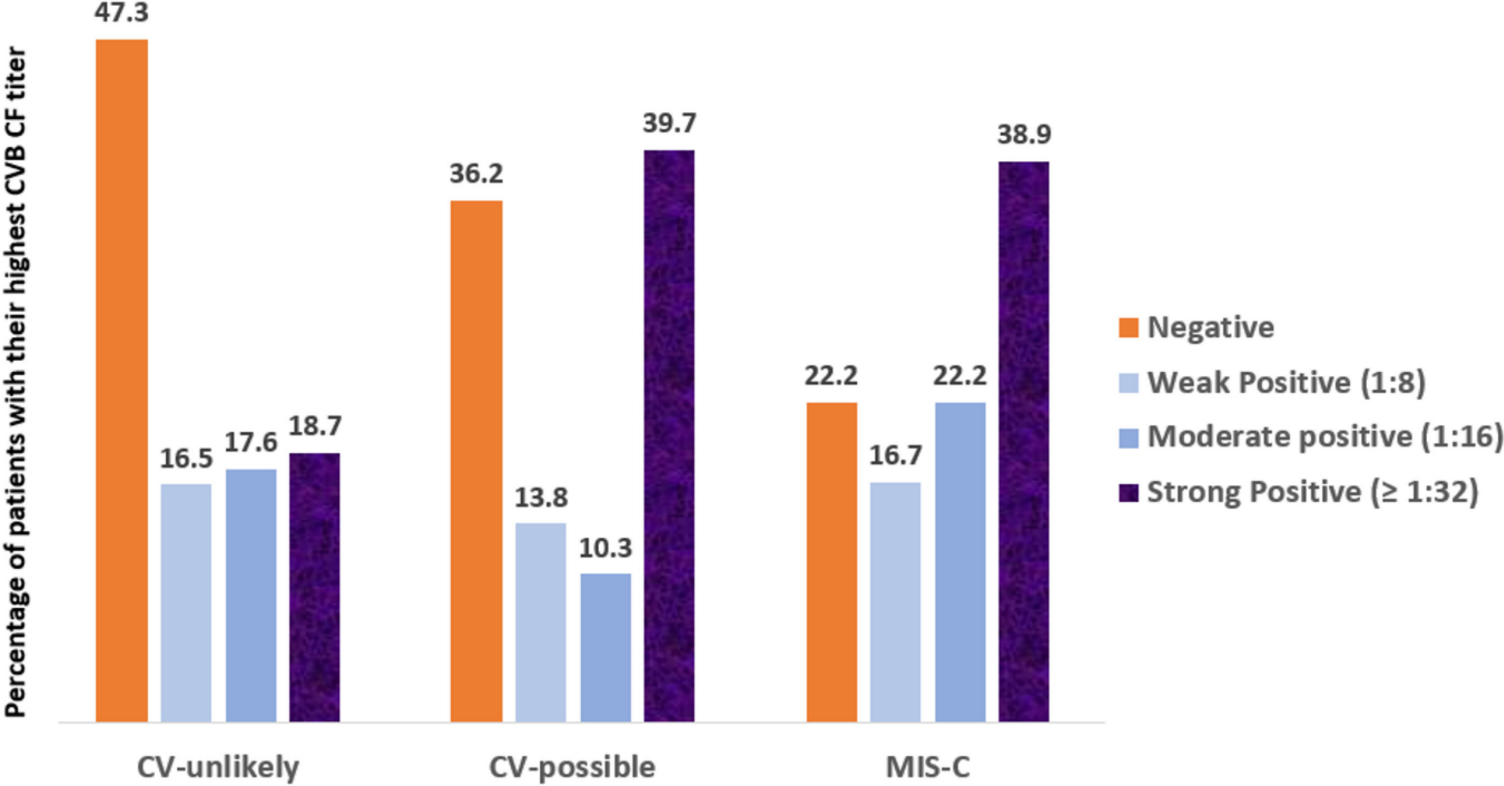
CVB CF positivity rate in regard to highest titer for serotypes B1–B6 among MIS-C (18 patients) versus CV-unlikely, excluding MIS-C (91 patients) and CV-possible (58 cases).

Overall, the likelihood of elevated CVB CF titers was relatively higher in MIS-C patients than non-MIS-C patients; however, the difference did not reach statistical significance (odds ratio: 2.07, 95% CI: 0.86–4.95, *P*: 0.10). When we categorized non-MIS-C patients into CV-unlikely and CV-possible groups, in comparing CVB CF testing results, MIS-C and CV-possible groups were generally indistinguishable (Fig. 5), although fewer MIS-C children had negative CVB CF titers (22.2% vs 36.2%). In comparison to CV-unlikely, both MIS-C and CV-possible comparisons revealed significant results. Comparing CV-possible to CV-unlikely by regression analysis showed a significant difference in the likelihood of elevated CVB CF titers (odds ratio: 1.65, 95% CI:1.06–2.56, *P*: 0.027). Strong CVB CF seropositivity (titer ≥1:32) was also significantly more frequent among CV-possible when compared with CV-unlikely (39.7% vs 18.7%; *P*: 0.005). Comparing MIS-C to CV-unlikely by regression analysis also showed significance (odds ratio: 1.92, 95% CI: 1.02–3.63, *P*: 0.04). The frequency of strong CVB seropositivity (titer ≥1:32) was also notably higher in MIS-C than CV-unlikely (38.9% vs 18.7%, *P*: 0.05) (Fig. 5).

There was no significant difference in the likelihood of higher CVA IFA titers between CV-possible and CV-unlikely groups (odds ratio: 1.17, 95% CI: 0.61–2.25, *P*: 0.61). The frequency of CVA strong seropositivity (titer: 1:1,600) also remained insignificant between the two groups (18/81, 22.2%, vs 17/52, 32.7%, p: 0.18). There was also no significant difference in the likelihood of CVA IFA titers between MIS-C versus CV-unlikely groups (odds ratio: 1.8, 95% CI: 0.76–4.65, *P*:0.17) (Fig. S1). The two groups also did not exhibit a significant difference in the frequency of CVA IFA strong seropositivity (5/17, 29.4%, 18/81, 22.2%, *p*: 0.52).

## DISCUSSION

In this study, we observed a significantly higher number of children with MIS-C with elevated CVB serological titers than those who were unlikely to have acute CV infection. Other significant findings include the association of race as a risk factor for CV illness. The higher rate of MIS-C in African Americans may imply a higher risk of MIS-C in this group or that prior CV infection may contribute to MIS-C. Additionally, much like many other viral infections (32–34), the pandemic significantly influenced CVB serological test positivity during the peak of social isolation in 2020.

Patients with MIS-C exhibited a significantly higher likelihood of elevated CVB CF titers than the control group. A two-hit theory has been proposed to explain MIS-C (10). This theory suggests that an initial SARS-CoV-2 infection triggers nonspecific polyclonal activation and proliferation of T cells and B cells. The second hit by another virus would activate this already enriched subpopulation of T cells and B cells, leading to hyperinflammation, autoantibodies, and tissue damage (10). CVs, and enteroviruses in general, offer an intriguing possibility to prime the immune system that would lead to MIS-C after a SARS-CoV-2 infection. Persistent CVB RNA remnants have been associated with chronic diseases (28, 29). Hypothetically, these remnants could trigger autoimmunity and hyperinflammation during recovery from SARS-CoV-2. The results of prior studies on the involvement of a secondary infection in the pathogenesis of MIS-C have been inconclusive. A study that globally addressed viral and bacterial specific transcripts, with emphasis on EBV and CMV transcripts, did not reveal significant results (11). Advanced phage immunoprecipitation sequencing technology, termed VirScan, which displays 93,904 epitopes from 206 human-infecting viruses, has been used to compare MIS-C with other groups in small studies (35). IgG antibodies to common viruses, such as respiratory syncytial virus (RSV), rhinovirus, herpesviruses, and enteroviruses, were shown in all children in the cohort (19 healthy children, 27 Kawasaki disease, 11 acute SARS-CoV-2, and 3 MIS-C cases) with no obvious MIS-C-associated infection (12). However, the small number of cases makes the interpretation of the results and comparison between cohorts challenging.

Our study reveals a high CV seropositivity even in the CV-unlikely group, suggesting widespread community transmission of the virus. This aligns with findings from other global studies (36–40). A retrospective study in Italy, involving 2,459 patients referred to a large academic hospital between 2004 and 2016, found seropositivity for at least one serotype in 69% of individuals when using neutralizing antibody assay method with a positive cut-off point at 1:32 (37).

Our study demonstrates a significantly higher strong CVB CF titers (≥1:32) and suggests a greater likelihood of elevated CVB CF titers among African American children than Whites, echoing findings with other viral infections, such as hepatitis B (41) and hepatitis C (42). These disparities may be attributable to socioeconomic factors or possible genetic differences. Consistent with our results, several studies have also found higher incidence of MIS-C among African Americans compared with Whites (43–46). There is yet to be a definitive explanation for this disparity. It is worth noting that in our study, a relatively higher frequency of MIS-C among African Americans, although statistically non-significant, coincided with a notably higher frequency of strong CVB CF titers in these patients. An intriguing proposal would be that African Americans’ higher risk of MIS-C is partially based on their higher incidence of recent CV preceding infection.

The adaptive immune response following Enterovirus infections is consistent with the general scheme of viral infections. Coxsackievirus-neutralizing IgM antibodies appear 3 days after infection, reach their peak on day 7, and typically vanish around 3 months after exposure. Anti-CV IgG antibodies released by adaptive B cells appear on day 4 after infection, reach their maximum level 2 to 3 weeks after exposure, and persist for years, although they may decrease gradually over time (47). Our study showed a significantly higher likelihood of elevated CVB CF titers in CV-possible than CV-unlikely groups, which suggests the higher specificity of the CF method in diagnosing acute or recent CV infections (within past few months) as compared with the IFA IgG assay. CF test produces a much stronger response to IgM than IgG but with less sensitivity can pick up IgG, as well (48–50). Specific children with repeat testing reviewed support that the CF antibodies are not permanent and can also show a rise to support an acute CV diagnosis. Particularly for the child with myocarditis, specific CV convalescent testing 4–8 weeks after the initial admission may have solidified a diagnosis. The seasonal variation and effect of the pandemic shown in this study also suggest that CF can support recent infection, as has been previously shown (51).

The 2020 decline in CVB CF seropositivity rate can be explained by implementation of strict hygiene measures, such as social distancing, mask wearing, and regular hand washing. We found a very low positivity rate for CVA CF test (2/16, 12.5%) in our community. This aligns with a recent study in the Western New York Community, with a CVA CF positivity rate of 2.3% among 257 patients (median age, 42 years, range 6–68 years) (51). This contrasts with the high positivity rate for CVA IFA IgG test. IgG by IFA testing may represent persistence of IgG from years before (52). This finding also explains why social distancing did not impact CVA IFA seropositivity rate during the pandemic. Furthermore, the same finding makes it challenging to assess any possible association between a recent CVA exposure and MIS-C development. Overall, the high CV IFA seropositivity in the general community can limit the effectiveness of CV IFA serological tests for documenting CV acute infection. The low accuracy of some enterovirus serological tests for diagnosis of acute infections has already been documented (36). For such tests, ideally, a paired acute and convalescent serological test with an interval of at least 4 weeks and a fourfold rise in serum titer is required to confirm an acute viral infection (36, 53, 54); however, obtaining such tests in clinical practice is challenging and usually has no treatment implication.

Our study has limitations. The serological test results are from hospitalized patients, so they may not accurately represent the community’s seroprevalence. To mitigate this bias, we categorized non-MIS-C cases into CV-possible and CV-unlikely, assuming that the CV seropositivity rate in the CV-unlikely group would more closely reflect that of the community. There is no available commercial molecular test specific for CVA and CVB, and most patients during this time did not have molecular testing done. Given that we only had a positive nasopharyngeal EV RT-PCR in a single patient, we were unable to correlate the CV serological tests with confirmatory tests. A serological test, such as IFA IgM, could have been helpful to distinguish recent infection and assist with interpretation of the CVA IgG IFA results. However, such a test has not been routinely performed during the study period. Additionally, we did not design this study to address how the cases of MIS-C varied in comparison to prospectively identified cases, so the association of CV can only be inferred. We had only access to patients’ files with MIS-C who had serological coxsackievirus test in their records. Therefore, we could not precisely assess the distribution of all admitted MIS-C patients through the years. The relatively small number of cases from this single institution with MIS-C impacted the power of the study.

### Conclusion

Our findings indicated a significantly higher likelihood of elevated CVB titers among patients with MIS-C, highlighting the need for larger scale studies to explore the role of secondary viral infections, including prior CVB in the development of MIS-C.

## SUPPLEMENTAL MATERIAL

Supplemental tables and figure spectrum.01741-24-s0001.docx Tables S1 to S8; Figure S1.

ASM does not own the copyrights to Supplemental Material that may be linked to, or accessed through, an article. The authors have granted ASM a non-exclusive, world-wide license to publish the Supplemental Material files. Please contact the corresponding author directly for reuse.
