# Supplementary material for: Serology supportive of recent coxsackievirus B infection is correlated with multisystem inflammatory syndrome in children (MIS-C)
Source: Microbiol Spectr. 2025 Feb 5;13(3):e01741-24. doi: 10.1128/spectrum.01741-24 (PMC11878073; doi:10.1128/spectrum.01741-24)
Supplement: Supplemental tables and figure — Tables S1 to S8; Figure S1. [file spectrum.01741-24-s0001.docx]

**Supplemental materials:**

**Table S1:** The recognized viral pathogens and the method of diagnosis among 182 admissions

| **Case*** | **Viral agent** | **Age (year)** | **Method of diagnosis** | **Prominent Clinical manifestation** | **Superimposed**  **bacterial infection** |
| --- | --- | --- | --- | --- | --- |
| 3 | HMPV^1^ | 7 | DFA^2^ | Pneumonia | Unspecified |
| 5 | EBV^3^ | 4 | PCR^4^ (Blood) | Pneumonia, Hepatitis | - |
| 16 | Parainfluenza 3 | newborn | DFA | URI^5^ | - |
| 21 | Enterovirus | 6 | PCR (nasopharyngeal) | Fever, skin rash on both hands, hand swelling | - |
| 27 | CMV^6^ | 2 | IgG, IgM positive | URI, exanthema | - |
| 38 | VZV^7^ | 15 | VZV IgG, IgM positive  PCR, skin lesion | Widespread Blisters, vesicles, red spots | - |
| 52 | EBV | 9 | PCR (blood) | Malaise, hepatitis, splenomegaly | - |
| 53 | Rhinovirus | 2 | RVP^8^ | Pneumonia | Unspecified |
| 59 | EBV | 14 | Heterophile antibody positive  Clinical course | Myositis, exanthema, Splenomegaly | - |
| 63 | HSV1^9^ | 1.5 | PCR,  oral mucous membrane | Oral Mucositis, Exanthema | - |
| 68 | Adenovirus | 4 | RVP | URI, malaise | - |
| 70 | Parvovirus | 1 | IgG positive  Clinical course | Intermittent fever, prolonged exanthema, loose stool | - |
| 82 | SARS-CoV-2 | 19 | PCR (NP^10^) | Malaise, body ache | - |
| 86 | EBV | 10 | IgM pos,  PCR (blood) | malaise, back pain, hepatitis, pneumonia | - |
| 87 | Parainfluenza 3 | 1.5 | RVP | URI, Myositis | - |
| 91 | EBV | 1 | IgG, IgM positive | Malaise, myalgia, splenomegaly | - |
| 93 | Rhinovirus | 1.5 | RVP | Pneumonia, respiratory failure | - |
| 97 | HMPV | 1.5 | RVP | Myocarditis, gastroenteritis | - |
| 109 | CMV | 8 | IgG IgM positive | URI, gastroenteritis | - |
| 117 | EBV | 2 | IgG, IgM positive | URI, gastroenteritis, exanthema | - |
| 118 | Adenovirus | 2 | RVP | Gastroenteritis, exanthema | - |
| 119 | SARS-CoV-2 | 8 months | PCR (NP) | Malaise, exanthema | - |
| 125 | Rhinovirus | 1.5 | RVP | URI, Pneumonia, myocarditis | - |
| 131 | CMV | 5 | IgG, IgM positive | URI, Myositis, gastroenteritis | - |
| 133 | SARS-CoV-2 | 8 months | PCR | Gastroenteritis | - |
| 134 | Parainfluenza 4 | newborn | RVP | URI | - |
| 143 | SARS-CoV-2 | 17 | PCR (NP) | Malaise, altered mental status | - |
| 148 | RSV^11^ | 3 months | RVP | pneumonia, exanthema, mucositis, sepsis | Unspecified |
| 159 | Adenovirus | 13 | RVP | gastroenteritis, exanthema | - |
| 161 | HMPV | 1.5 | RVP | URI, Pneumonia | - |
| 162 | HMPV | 1.5 | RVP | URI, conjunctivitis, gastroenteritis | - |
| 165 | HMPV | 6 | RVP | Pneumonia | Unspecified |
| 167 | EBV | 5 | IgM positive  PCR (Blood) | URI, mucositis, abdominal pain, | Sepsis, Streptococcus Pyogenes |
| 171 | HMPV | 2 | RVP | URI, Pneumonia, exanthema | - |
| 172 | HSV1 | 5 | PCR, mucosal | Oral mucositis, exanthema | - |
| 173 | Adenovirus | 2 | RVP (PCR) | Widespread exanthema, | - |
| 176 | SARS-CoV-2 | 18 | PCR (NP) | URI, pneumonia, respiratory failure, cardiogenic shock | - |

****: Case numbers are sorted according to the date of admission.***

***Abbreviations****: 1; Human metapneumovirus, 2; Direct Fluorescent Antibody, 3; Epstein-Barr Virus, 4; Polymerase Chain Reaction, 5; Upper Respiratory Tract Infection, 6; Cytomegalovirus, 7; Varicella Zoster Virus, 8; Respiratory Viral Panel (Panther Hologic Fusion method), 9: Herpes Simplex Virus type1, 10; Nasopharyngeal, 11; Respiratory Syncytial Virus*

**Table S2**: Cases with bacterial infections among 182 admissions. 8 patients had concomitant viral infection.

| **Case*** | **Bacterial pathogen** | **Age (year)** | **Method of diagnosis** | **condition** | **Concomitant viral infection** |
| --- | --- | --- | --- | --- | --- |
| 1 | Staphylococcus Aureus | 18 | Wound culture | Buttock abscess | Unspecified |
| 3 | Unspecified | 7 | - | pneumonia | HMPV^1^ |
| 4 | Unspecified | 8 | - | Cholangitis | - |
| 12 | MSSA^2^ | 1 | Blood culture | Septic shock | - |
| 20 | Unspecified | newborn | - | Neonatal sepsis | - |
| 24 | MRSA^3^ | 7 | Tracheal Culture | Pneumonia | Unspecified |
| 28 | Mycoplasma | 16 | Serology | Oral mucositis | - |
| 41 | Mycoplasma | 17 | Serology | Pneumonia | - |
| 47 | Gram positive Bacilli | 17 | Blood culture | Sepsis | - |
| 72 | Unspecified | 13 | - | Endocarditis | - |
| 85 | Mycoplasma | 14 | Serology | Pneumonia/pericarditis |  |
| 96 | Unspecified | 2 | - | Acute sinusitis/peri-orbital Cellulitis | - |
| 101 | Pneumococcus | 1.5 | Blood culture | Sepsis | - |
| 110 | Staphylococcus Aureus | 6 | Blood culture | Septic shock |  |
| 125 | Unspecified | 1.5 | - | Pneumonia | Rhinovirus |
| 135 | Klebsiella | 2 mo^4^ | Blood culture | Meningitis | - |
| 137 | Mycoplasma | 15 | serology | Guillain Barre | - |
| 139 | Unspecified | 12 | - | Chemotherapy induced Sepsis | - |
| 140 | Mycoplasma | 10 | Serology | MIRM^5^ | - |
| 141 | Unspecified | 5 | - | Pyelonephritis | - |
| 145 | Unspecified | 18 | - | Sepsis/atypical HUS^6^ | - |
| 148 | Unspecified | 3 mo | - | Pneumonia | RSV^7^ |
| 153 | unspecified | 6 | - | Cervical Lymphadenitis | - |
| 154 | Unspecified | 2 | - | Pneumonia | Unspecified |
| 155 | Staphylococcus aureus | 15 | Blood culture | TSS^8^ | - |
| 164 | Mycoplasma | 14 | Serology | Oral mucositis | - |
| 165 | Unspecified | 6 | - | Pneumonia | HMPV |
| 167 | Streptococcus pyogenes | 5 | Blood culture | Sepsis | EBV |
| 169 | Mycoplasma | 5 | serology | Meningoencephalitis | - |

****: Case numbers are sorted according to the date of admission.***

***Abbreviations:*** *1; Human metapneumovirus, 2; Methicillin Sensitive Staphylococcus Aureus, 3; Methicillin Resistant Staphylococcus Aureus, 4; month old, 5; Mycoplasma pneumoniae-Induced Rash and Mucositis, 6; Hemolytic Uremic Syndrome, 7; Respiratory syncytial virus; 8; Toxic shock syndrome.*

**Table S3**: non-infectious conditions excluding MIS-C among 182 admissions

| **Case*** | **condition** | **Age**  **(year)** | **Clinical primary presentation** |
| --- | --- | --- | --- |
| 6 | Atypical Kawasaki Disease | 3 | Fever, oral mucositis, abdominal pain, skin rash |
| 8 | Sickle cell crisis | 20 | Chest pain, SOB^1^, Hip pain |
| 10 | SLE^2^ pericarditis | 13 | Chest pain, palpitation, SOB |
| 11 | Pyruvate carboxylate deficiency | 1 | Lethargy, SOB, failure to thrive |
| 13 | Cardiogenic shock caused by SVT^3^ | newborn | Lethargy, palpitation, SOB |
| 15 | Prolonged High ostomy output with unrecognized cause, Hx of ostomy, | 6 | abdominal pain, nausea |
| 17 | B cell acute lymphocytic leukemia, GVHD^4^ | 7 | Fever, Headache, nausea, diarrhea, pallor |
| 25 | Kawasaki Disease | 3 | Fever, conjunctivitis, skin rash, foot edema |
| 30 | SJS^7^ unknown cause | 9 | Sore throat, gingivitis, oral ulcers, Skin rash |
| 31 | ADEM^8^ | 9 | Fever, left eye blurry vision, ataxia |
| 37 | Acute pancreatitis due to HG^9^ | 11 | Fever, abdominal pain, nausea, vomiting |
| 39 | ADEM | 12 | Fatigue, blurry vision, ataxia, skin rash |
| 43 | SJS/ EM^10^ unspecified etiology | 10 | Oral lesions, erythematous skin rash |
| 44 | Adrenal neuroblastoma | 2 | Opsoclonus myoclonus |
| 46 | Pneumonia due to vaping | 18 | Respiratory distress, chest pain, weight loss |
| 51 | Child abuse, subdural hematoma | 5 | Seizure |
| 54 | Drug abuse, Rhabdomyolysis | 13 | Altered mental status, Palpitation |
| 55 | Self-inflicted injury, history of Autism | 14 | Fascial swelling, dark circle under eyes |
| 60 | SLE carditis | 13 | Chest pain, palpitation, SOB |
| 64 | Dilated cardiomyopathy, unknown cause | 4 months | Respiratory distress, shock |
| 76 | Fever following Vaccination | 4 months | Fever, decreased appetite, loose stool |
| 78 | Myocarditis associated Covid Vaccination | 15 | Chest pain, palpitation |
| 79 | Idiopathic Itching Hives | 4 | Urticaria |
| 80 | Myocarditis associated Covid Vaccination | 16 | Chest pain, palpitation |
| 81 | Myocarditis associated Covid Vaccination | 14 | Chest pain, palpitation |
| 84 | Paraspinal CSF leak following surgery | 17 | Chest pain, palpitation, SOB |
| 92 | Atypical Kawasaki Disease | 2 | Fever, conjunctivitis, cracked lips, diarrhea |
| 94 | Fetal growth restriction in pregnancy | 18 | Fetal growth restriction in 24w of pregnancy |
| 103 | Giant cell hepatitis/ combs positive hemolytic anemia | 3 months | Irritability, jaundice, pallor |
| 105 | DRESS^11^syndrome due to medication | 10 | Fever, malaise, blurry vision, confusion, skin rash |
| 124 | Kawasaki Disease | 2 | Fever, conjunctivitis, cracked lips, skin rash |
| 126 | Atypical Guillain Barre (Miller-Fischer type), unknown cause | 10 | Lower extremity weakness, blurry vision, urinary incontinence |
| 129 | HLH associated with anaplastic lymphoma | 19 | Fever, abdominal pain, malaise, diarrhea |
| 146 | Dermal hypersensitivity | 6 | Bullous skin lesions in lower extremity |
| 157 | Serum sickness | 6 | Fever, joint pain, skin rash, diarrhea |
| 158 | Atypical Kawasaki Disease | 3 | Fever, skin rash, cervical lymphadenopathy |
| 160 | Atypical Kawasaki Disease | 2 | Fever, conjunctivitis, cracked lips, skin rash |
| 168 | Serum sickness | 6 | Low-grade fever, Bilateral Knee and right elbow joint pain and swelling, skin rash |
| 177 | Periodic fever syndrome | 1.5 | Intermittent fever for 6 months, pallor |
| 178 | Kawasaki Disease | 5 | Fever, skin rash, conjunctivitis, strawberry tongue, cervical adenopathy |
| 179 | Lupus carditis | 19 | Chest pain, palpitation, SOB |
| 180 | Allergic Hives | 15 | Persistent urticaria |
| 181 | Migraine with Bell’s palsy | 20 | Headache, nausea, left fascial drooping |
| 182 | Head & abdominal trauma | 4 | Altered mental status, transaminitis |

****: Case numbers are sorted according to the date of admission.***

***Abbreviations****: 1; Shortness of breath, 2; Systemic lupus erythematosus, 3; Supra ventricular tachycardia, 4; Graft-versus-host disease, 5; Hemophagocytic Lympho-histiocytosis, 6; Juvenile Rheumatoid Arthritis, 7; Stevens-Johnson syndrome, 8; Acute Disseminated Encephalomyelitis, 9; Hypertriglyceridemia, 10; Erythema Multiform, 11; Drug Rash with**Eosinophilia and Systemic Symptoms.*

**Table S4**: Cases with available enterovirus Polymerase Chain Reaction (EV-PCR) on nasopharyngeal and or cerebrospinal fluid (CSF) specimens.

| **Case*** | **Age (years), Sex** | **EV^1^ PCR^2^**  **Np^3^** | **EV PCR**  **CSF^4^** | **Other Viruses**  **CSF PCR**  **(all tested negative)** | **Presentation**** | **Final**  **diagnosis** | **CVB^5^ CF^6^ maximum titer** | **CVA^7^ technique**  **Maximum titer** |
| --- | --- | --- | --- | --- | --- | --- | --- | --- |
| 7 | 1, M^8^ | Neg^9^ | - | ^-^ | URI^13^, skin rash | Unspecified Viral syndrome | neg*** | CF+, neg |
| 11 | 1, F^14^ | - | Neg | CMV^10^, EBV^11^, HSV^12^1/2 | SOB^15^, Lethargy | Carboxylate deficiency | neg | CF, neg |
| 16 | NB^16^, M | Neg | - | - | URI | Parainfluenza 3  infection | - | CF, neg |
| 18 | 10, M | - | Neg | HSV1/2 | Cough, lethargy,  Muscle pain | Hx^17^ of JRA^18^ & MAS^19^  Superimposed unspecified viral infection | B1, B5  1:32 | CF, neg |
| 20 | NB, M | Neg | Neg | HSV1/2 | SOB, unresponsiveness | Bacterial Sepsis | neg | CF, neg |
| 21 | 6, M | **Pos^20^** | **-** | - | Skin rash, hand swelling | Enteroviral infection | neg | IFA^21^, all tested serotypes++  1:800 |
| 23 | 3, M | - | Neg | viral encephalitis Panel+++ | Lower extremity weakness | Viral infection induced  Acute flaccid myelitis | B5, 1:32 | - |
| 24 | 7, M | Neg | - | - | Cough, muscle pain | Viral pneumonia,  Superimposed MRSA bacterial infection | B1, 1:8 | A7, A16  1:1600 |
| 26 | 17, M | Neg | Neg | HSV1/2 | Headache, confusion, photophobia | Unspecified viral infection | B1, B2, B4, B5  1:8 | IFA, neg |
| 28 | 16, M | Neg | - | - | Oral mucositis | Mycoplasma induced  Oral mucositis | B1, B4, B6  1:16 | IFA, neg |
| 31 | 6, M | - | Neg | HSV1/2 | Blurry vision, photophobia, Ataxia | ADEM^22^ | Neg | IFA, A16  1:1600 |
| 32 | 6, F | neg | - | - | Sore throat, skin rash | Unspecified viral infection | B1, B4, B5, B6  1:32 | IFA, all tested serotypes  1:1600 |
| 36 | 11, F | - | Neg | - | Headache, Myalgia, confusion  Skin rash | Unspecified viral syndrome | B5, B6  1:8 | IFA, A9, A16, A 24  1:800 |
| 39 | 12, F | - | Neg | HSV1,2 | Headache, Blurry vision, Ataxia | ADEM | - | IFA, neg |
| 40 | 2, F | - | Neg | Viral encephalitis panel | Seizure | Viral Febrile seizure | Neg | IFA, all serotypes  1:100 |
| 41 | 17, F | Neg | - | - | Cough, nasal congestion | Mycoplasma pneumonia | B4, B5  1:32 | IFA, A7, A9 A16  1:800 |
| 44 | 2, F | - | Neg | HSV1/2 | Opsoclonus myoclonus | Adrenal neuroblastoma,  Chiari I malformation | Neg | IFA, all serotypes  1:400 |
| 49 | 4, F | Neg | - | - | Skin rash, conjunctivitis, | Unspecified viral syndrome | B5, 1:64 | IFA, A16 A24  1:1600 |
| 54 | 13, F | - | Neg | HSV1/2 | Tachycardia, altered mental status | Hx of autism, Rhabdomyolysis  Intentional drug overdose | B5, 1:8 | IFA, all serotypes  1;800 |
| 57 | 1, M | Neg | - | - | Skin rash | Unspecified viral syndrome | Neg | IFA, A7, A24  1:400 |
| 77 | 17, M | Neg | - | - | Headache, vomiting | Unspecified viral meningitis | Neg | IFA, all serotypes  1:800 |
| 78 | 15, F | Neg | - | - | Chest pain, tachycardia | Myocarditis associated with covid vaccine | Neg | IFA, all serotypes  1:1400 |
| 79 | 4, M | Neg | - | - | No fever,  Itching Hives | Acute urticaria | B6, 1:8 | IFA, all serotypes  1:1600 |
| 81 | 14, M | Neg | - | - | Chest pain, tachycardia | Myocarditis associated with covid vaccine | B5, 1:8 | IFA, all serotypes  1:400 |
| 87 | 1.5, F | Neg | - | - | Lower extremity weakness | Parainfluenza 3 viral myositis | Neg | IFA, all serotypes  1:800 |
| 93 | 1.5, M | Neg | - | - | Nasal congestion, SOB | Rhinovirus viral infection | - | IFA, all serotypes  1:800 |
| 96 | 2, M | Neg | - | - | Right eye swelling, nasal discharge | Bacterial sinusitis, Peri-orbital cellulitis | Neg | IFA, all serotypes  1:1600 |
| 98 | NB, F | - | Neg | EBV, CMV, HSV1/2 | Altered mental status, skin rash | Unspecified viral infection | - | IFA, all serotypes  1:1600 |
| 100 | 3, M | - | Neg | HSV1/2 | Altered mental status, skin rash | Unspecified viral infection | B4, B5  1:16 | IFA, all serotypes  1:1600 |
| 101 | 1.5, M | - | Neg | HSV1/2 | Lethargy | Pneumococcal sepsis | B1, B6  1:8 | IFA, all serotypes  1:800 |
| 105 | 10, M | - | Neg | HSV1/2 | Headache, seizure, altered mental status, skin rush | DRESS^23^ syndrome secondary to medication | B1, B4, B5  1:16 | IFA, all serotypes  1:1600 |
| 106 | 11, M | - | Neg | EBV, CMV, HSV1/2 | Altered mental status | Unspecified viral encephalitis | Neg | IFA, neg |
| 109 | 8, M | Neg | - |  | Pharyngitis, emesis, diarrhea | CMV infection | - | IFA, all serotypes  1:100 |
| 110 | 6, F | - | Neg | Viral encephalitis panel | Headache, altered mental status, skin rash | Staphylococcus aureus septic shock | - | IFA, all serotypes  1:200 |
| 123 | 9, F | Neg | - | - | Chest pain, SOB, cardiogenic shock | Unspecified Viral myocarditis | Neg | IFA, A7, A16  1:800 |
| 130 | 13, M | Neg | - | - | Oral mucositis, skin rash | Unspecified viral infection | B1, B2, B4, B6  1:64 | IFA, neg |
| 137 | 15, M | Neg | - | - | Blurry vision, fascial weakness,  cough | Miller-Fischer variant Guillain Barre secondary to mycoplasma | B5  1:64 | IFA, all serotypes  1:1600 |
| 148 | 3 mo^24^, Male | - | Neg | HSV1/2 | Altered mental status, cough, emesis, conjunctivitis, skin rash | RSV^25^ viral pneumonia,  Superimposed bacterial sepsis | Neg | IFA, A24  1:400 |
| 150 | 8, F | - | Neg | CMV, HSV1/2, Human herpesvirus 6, Parechovirus, VZV | Altered mental status, jerking movements | Unspecified viral encephalitis  (CSF viral panel negative) | B6,  1:32 | IFA, A7, A9, A24  1:1600 |
| 172 | 5, F | Neg | - | - | Skin rash, oral mucositis | HSV^26^1 infection | Neg | IFA, neg |
| 174 | 14, F | Neg | - | - | Fatigue, joint pain, emesis | HLH^27^ induced by a viral infection | B1, B2, B6  1:256 | IFA, neg |
| 181 | 20, F | Neg | Neg | HSV1/2, EBV | No fever, Headache, left eye Blurry vision, fascial palsy | Migraine, Bell’s palsy | Neg | IFA, all serotypes  1:400 |

****: Case numbers are sorted according to the date of admission.***

**: All cases had fever at presentation unless specified otherwise. ***: negative in all tested serotypes (complement fixation < 1:8, Immunofluorescence assay < 1:100).

+: Coxsackievirus A complement fixation test on A2, A4, A7, A9, A10, A16.

++: All CVA serotypes tested through IFA including A7, A9, A16, A24.

+++: Viral encephalitis panel (PCR): Adenovirus, West Nile virus, CMV, enterovirus, Herpes Virus ½, Varicella Zoster virus, Epstein Barr virus, Human Herpes Virus 6, Eastern equine encephalitis, Saint Louis encephalitis virus, enterovirus.

***Abbreviations****: 1; Enterovirus, 2; Polymerase Chain Reaction, 3; nasopharyngeal, 4; Cerebrospinal fluid, 5; coxsackievirus B, 6; complement Fixation, 7; coxsackievirus A, 8; male, 9; negative, 10; Cytomegalovirus , 11; Epstein Barr Virus, 12; Herpes Simplex Virus, 13; Upper Respiratory Infection, 14; Female, 15; Shortness of breath, 16; Newborn, 17; History, 18; Juvenile Rheumatoid arthritis, 19; Macrophage Activating Syndrome, 20; Positive, 21; Immunofluorescence assay, 22; Acute Disseminated Encephalomyelitis, 23; Drug reaction with eosinophilia and systemic symptoms, 24; month-old, 25; Respiratory Syncytial Virus, 26; Herpes Simplex Virus, 27; Hemophagocytic lymph histiocytosis.*

**Table S5**: Characteristics of three patients who were re-admitted.

| **Case*** | **Admission interval**  **(months)** | **Clinical presentation,**  **remarkable lab findings,**  **Final diagnosis**  **(1^st^ admission)** | **CVB^1^ CF^2^,**  **Results**  **(1^st^ admission)** | **CVA^3^ IFA^4^ IgG**  **results**  **(1^st^ admission)** | **Clinical presentation,**  **remarkable lab findings,**  **Final diagnosis**  **(2nd admission)** | **CVB CF**  **results**  **(2nd admission)** | **CVA IFA IgG results**  **(2nd admission)** |
| --- | --- | --- | --- | --- | --- | --- | --- |
| 33, 37 | 3 | 5 yo^5^ female, Intermittent fever, cough, maculopapular skin rash.  CMV^6,^ EBV^7^, influenza, parainfluenza, mycoplasma serology/PCR^8^ negative  **Final Dx^9^:** unspecified viral syndrome | **CVB CF:**  B1, B2, B3, B4, B6 < 1:8 (negative)  B5: 1:16 | **CVA IFA IgG:**  A7, A9, A16, A24  < 1:100 (negative) | Fever, Severe abdominal pain, vomiting.  TG^10^: 6780 mg/dL, amylase: 181 U/L, lipase: 323 U/L  **Final Dx:** Acute pancreatitis due to hyperglyceridemia | **CVB CF:**  B1, B2, B4, B5, B6: 1:16,  B3: 1:32 | **CVA IFA IgG:**  A7, A9, A16, A24  < 1:100 (negative) |
| 30, 43 | 32 | 9 yo boy, gingivitis, cold sore, sore throat, and penile rash. No significant fever.  CMV, EBV, HSV^11^1 and mycoplasma all negative.  **Final Dx**: SJS^12^ | **CVB CF:**  B1, B2, B3: 1:8, B4, B5: 1:32; B6 1:16. | **CVA IFA IgG:**  A7, A9, A24: 1:100;  A16,1:200 | Genital and oral lesions, nasal congestion, sore throat, and a lesion on the nose bridge.  CMV, EBV, HSV1, HSV2, and mycoplasma negative. HSV1/2 PCR negative on a mouth lesion  **Final Dx**: Recurrent EM^13^/SJS | **CVB CF:**  All serotypes negative  serotypes all turned negative | **CVA IFA IgG:**  A7, A9, A24: 1:100;  A16,1:200 |
| 45, 176 | 37 | 15-year-old female, with scleroderma, admitted with generalized body pain, SOB^14^ palpitations, and extremity cyanosis.  WBC^15^ 13.8 and ANC^16^ of 9, troponin was 9 ng/mL. Echocardiogram showed an LVEF of 40%.  **Final Dx:** acute viral myocarditis with an unspecified pathogen. | **CVB CF:**  All serotypes negative.  . | **CVA IFA IgG:**  All serotypes negative | Fever, headache, chest pain, shortness of breath, and a purple rash. She developed respiratory failure, was intubated, and went into cardiogenic shock.  A PCR test for RVP^17^ came back negative, but a PCR test for SARS-CoV-2 came back positive.  **Final Dx**: acute Covid infection. | **CVB CF:**  B2: negative  B1, B3, B4, B6: 1:8, B5: 1:16 | **CVA IFA IgG:**  A7, A9, A16: 1:100; A24: 1:400 |

****: Case numbers are sorted according to the date of admission.***

***Abbreviations:*** *1; coxsackie virus B, 2; complement fixation, 3; coxsackie virus A, 4; Indirect immunofluorescence assay, 5; years old, 7: cytomegalovirus, 7; Epstein Barr-virus, 8; polymerase chain reaction, 9; diagnosis, 10; Triglyceride, 11; Herpes simplex virus, 12; Stevens- Johnson Syndrome, 13; Erythema Multiforme, 14; Shortness of breath, 15; white blood cell, 16; absolute neutrophil count, 17; Respiratory Viral Panel.*

**Table S6:** Clinical and laboratory characteristics of 19 MIS-C cases.

| Case* | Age (year), Sex, Race | Temp^1^  (MAX) | CRP  mg/L | Cardiac involvement | | | | Mucocutaneus  involvement | GI^4^ | Shock | neurological | Hematological  Plt^5^-ALC^6^  (*10^3^/mm^3)^ | | Coagulopathy | | |
| --- | --- | --- | --- | --- | --- | --- | --- | --- | --- | --- | --- | --- | --- | --- | --- | --- |
|  |  |  |  | LVEF^2^  % | Coronary artery abnormality | Troponin  (ng/ml) | BNP^3^  Pg/ml |  |  |  |  | Plt | ALC | PT^7^ | PTT^8^ | D-dimer  (mg/L) |
| 56 | 6, M^9^, A^10^ | 39.3 | 306 | 69.7 | - |  |  | Conjunctivitis | 🗹 | - | - | 183 | 1.1 | 13.8 | ***46.6*** | ***1.88*** |
| 58 | 6, F^11^, W^12^ | 38.6 | 62 | 63.0 | - | <0.01 |  | Rash | 🗹 | - | Headache | 165 | ***.4*** | ***15.6*** | ***38.4*** | ***1.49*** |
| 61 | 9, M, A | 38.9 | 33 | 63.7 | - | <0.01 | <10 | - | 🗹 | - | Headache | ***138*** | ***0.9*** |  | 29.4 | ***3.51*** |
| 62 | 6, M, A | 38.9 | 138 | ***48.1******* | - | ***0.24*** | ***>5000*** | Rash | 🗹 | 🗹 |  | 247 | 1.3 | ***15.2*** | ***38.1*** | ***2.76*** |
| 65 | 3, M, W | 38.5 | 159 | 70.7 | - | <0.01 | ***1306*** | Conjunctivitis-cracked lips | 🗹 | - | Headache | ***110*** | ***.8*** | 13.7 | ***35.2*** | ***4.72*** |
| 66 | 16, M, W | 39 | 55 | 62.0 | - | <0.05 | <10 | Rash-mucositis | 🗹 | - | - | ***29*** | 1.1 | 12.9 | 23.7 | <0.27 |
| 73 | 12, M, W | 39.2 | 233 | 69.0 | - | <0.05 | 84 | Conjunctivitis-mucositis | 🗹 | 🗹 | Headache | 161 | 1.1 | ***17.4*** | ***41.0*** | ***1.62*** |
| 74 | 4, F, A | 39.4 | 387 | 58.7 | - |  | ***1272*** | - | 🗹 | 🗹 | - | 297 | ***.7*** | 14.6 | 34.1 | ***3.61*** |
| 75 | 9 mo^13^, M, A | 38.5 | 227 | 70.4 | - |  | 686 | Mucositis | 🗹 | - | - | 141 | 1.8 |  |  | ***1.72*** |
| 99 | 8, F, A | 38.5 | 49 |  |  | ***0.68*** | ***166*** | Fascial swelling | 🗹 | - | Headache | 145 | 1.4 | 13.3 | 25.5 | ***1.56*** |
| 104 | 10, M, A | 38.9 | 329 | ***49.8*** | - | ***1.06*** | ***464*** | - | - | 🗹 | Headache  Confusion | 258 | 1.2 | ***16.0*** | ***36.9*** | ***2.08*** |
| 107 | 5, M, W | 39.5 | 99 | ***54.9*** | - | ***0.08*** | ***501*** | Rash | - | - | - | 163 | 1.0 | ***17.5*** | 33.8 | ***3.52*** |
| 111 | 6, F, W | 39.6 | 132 |  | - | <0.01 | 25 | - | 🗹 | - | Headache, lethargy | 235 | 2.8 | ***15.6*** | ***41*** | 0.28 |
| 112 | 4, F, W | 39.2 | 170 | 67.4 | - | 0.03 | ***1098*** | - | 🗹 | - | Headache  Lethargy | ***80*** | ***.8*** | ***15.8*** | ***40.5*** | ***2.6*** |
| 113 | 17, M, W | 38.6 | 270 | 60.4 | - | 0.04 | ***279*** | Rash-Conjunctivitis-Mucositis | 🗹 | - | - | ***102*** | ***.7*** | ***16.7*** | ***44.4*** | ***4.04*** |
| 114 | 7, M, A | 38.7 | 45 | 67.4 | - | 0.02 | 21 | Rash-mucositis | - | - | - | 167 | 1.4 | 13.6 | ***41.6*** | ***3.64*** |
| 115 | 7, M, W | 39.5 | 152 | 55.7 | - | ***0.11*** | ***111*** | Rash-Conjunctivitis | 🗹 | - | - | 156 | 1.0 | ***16.5*** | 34.2 | ***0.7*** |
| 120 | 10, F, W | 38.9 | 130 | 71.8 | - | <0.01 | 17 | Rash-mucositis | 🗹 | - | - | ***83*** | ***.2*** | ***16.4*** | ***35.6*** | ***2.25*** |
| 122 | 5, M, W | 39.1 | 56 | 79.6 | - | <0.01 | <10 | Conjunctivitis-mucositis | 🗹 | - | - | 311 | ***.9*** | ***17.0*** | 34.0 | ***1.97*** |

****: Case numbers are sorted according to the date of admission.***

**: Italic Bold values indicate abnormal levels.

***Abbreviation:*** *1; Temperature, 2; Left ventricular ejection fraction, 3; Brain natriuretic peptide, 4; Gastrointestinal, 5; Platelet, 6, Absolute Lymphocyte Count, 7; Prothrombin Time, 8; Partial Thromboplastin Time 9; Male, 10; African American, 11; Female, 12; White, 13; month-old*

**Table S7**: laboratory findings in 19 cases diagnosed with MIS-C

| Laboratory test | Mean | Median | Min^1^ | Max^2^ | SD^3^ |
| --- | --- | --- | --- | --- | --- |
| **Inflammatory Markers:**  CRP^4^ (mg/L)  ESR^5^ (mm/h)  Procalcitonin (ng/ml)  Ferritin (ng/ml) | 159.8  43.9  6.5  836.5 | 138  37.5  3.4  465 | 33.6  10  0.05  145 | 387  120  51  3734 | 106  25.7  11.8  995.5 |
| **Cardiac markers:**  Troponin (ng/ml)  BNP6 (pg/ml) | 0.15  690.6 | 0.02  222 | 0.01  10 | 1.06  5000 | 0.29  1237 |
| **Hematological findings**:  ANC^7^ (×10^3^/mm^3^)  ALC^8^ (×10^3^/mm^3^)  Platelet (10^3^/mm^3^)  Hemoglobin (10^3^/mm^3^) | 7.9  1.08  166.7  11.5 | 7.2  1  161  11.5 | 3.3  0.2  29  7.3 | 18.7  2.8  311  18.1 | 3.74  0.55  74.8  2.36 |
| **Coagulation Markers:**  PT^9^ (seconds)  PTT^10^ (seconds)  INR^11^  D-dimer (mg/L)  Fibrinogen (mg/dL) | 15.5  36.1  1.24  2.42  471.1 | 15  36.2  1.23  2.17  479 | 12.9  23.7  0.96  0  254 | 19.6  46.6  1.7  5  711 | 1.74  6.1  0.18  1.18  128.3 |

***Abbreviations:*** *1: minimum, 2: Maximum, 3: Standard Deviation, 4: C-reactive protein, 5: Erythrocyte sedimentation rate, 6: Brain natriuretic peptide, 7: Absolute neutrophil count, 8: Absolute lymphocyte count, 9: Prothrombin time, 10: Partial thromboplastin time, 11: International normalization ratio.*

Table S8: The results of CVB CF and CVA IFA IgG in 19 patients with MIS-C

| **Case*** | **CVB CF^1^**  **Positive results** | **CVA IFA^2^ IgG**  **results** |
| --- | --- | --- |
| 56 | **B1-B6: neg**** | - |
| 58 | B5: 1:16  B1: 1:8 | A7, A9, A16, A24: 1:1600 |
| 61 | B1, B5 :1:32,  B6: 1:16,  B2: 1:8 | - |
| 62 | B5: 1:64,  B2, B3: 1:16,  B1, B4, B6: 1:8 | A7, A9, A16, A24: 1:1600 |
| 65 | B1, B3, B5, B6: 1:8 | A7, A9, A16, A24: 1:400 |
| 66 | **B1-B6: neg** | A7, A9, A16, A24: 1:1600 |
| 73 | B2: 1:32  B6: 1:8 | A7, A9, A16, A24: 1:1600 |
| 74 | B5: 1:8 | A7, A9, A16, A24: 1:400 |
| 75 | B5: 1:16 | - |
| 99 | B5: 1:32  B2, B3, B4, B6: 1:16  B1: 1:8 | A7, A9, A16, A24: 1:1600 |
| 104 | B3: 1:16 | A7, A9, A16, A24: 1:800 |
| 107 | B3: 1:32  B5, B6: 1:16  B2:1:8 | A7, A9, A16, A24: 1:400 |
| 111 | B4, B5: 1:32  B6: 1:16  B2: 1:8 | A7, A9, A16, A24: 1:400 |
| 112 | B2, B3, B4, B5, B6: 1:8 | A7, A9, A16, A24: 1:400 |
| 113 | - | A7, A9, A16, A24: 1:800 |
| 114 | B2, B5: 1:64  B1, B3, B4, B6: 1:32 | A7, A9, A16, A24: 1:800 |
| 115 | **B1-B6: neg** | A7, A9, A16, A24: 1:200 |
| 120 | **B1-B6: neg** | A16, A24: 1:100 |
| 122 | B4, B6: 1:16  B1, B5: 1:8 | A7, A9, A16, A24: 1:800 |

****: Case numbers are sorted according to the date of admission.***

**: Negative results are Bold.

*Abbreviations: 1; Complement fixation, 2: Indirect immunofluorescence assay*


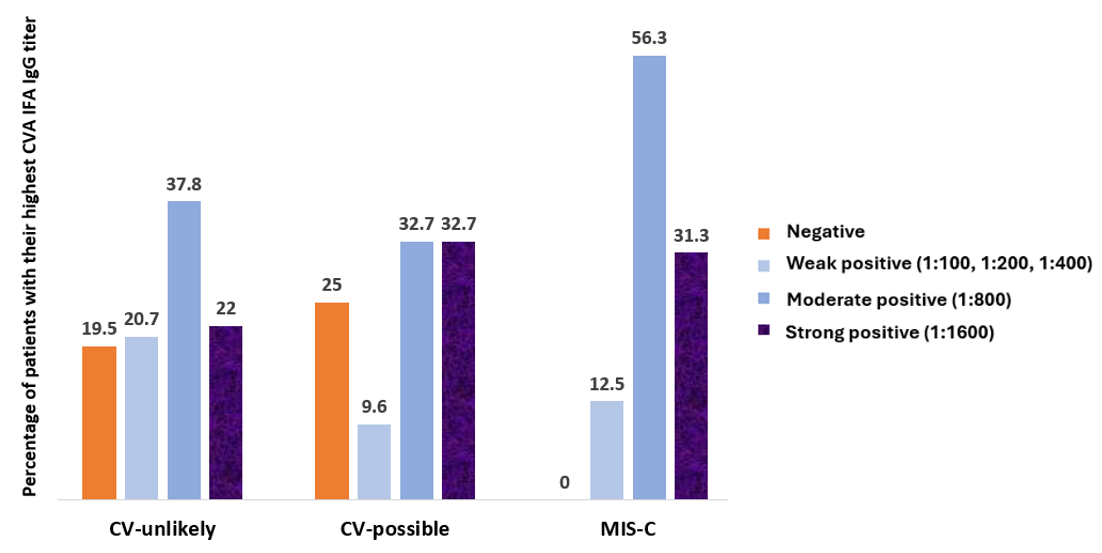


**Figure S1**: CVA IFA IgG positivity rate regarding highest titer for serotypes A7, A9, A16, and A24 among MIS-C (16 patients), CV-unlikely (82 patients), and CV-possible (52 cases). There was no significant difference in the likelihood of CVA IFA titers between MIS-C versus CV-unlikely groups (odds ratio: 1.8, 95% CI: 0.76-4.65, *p*-value:0.17).
